# Supplementary material for: A genome-wide CRISPR screen identifies GRA38 as a key regulator of lipid homeostasis during Toxoplasma gondii adaptation to lipid-rich conditions
Source: Nat Commun. 2025 Dec 17;16:11177. doi: 10.1038/s41467-025-66137-5 (PMC12711892; doi:10.1038/s41467-025-66137-5)
Supplement: Supplementary file 1 — Supplementary Information File [file 41467_2025_66137_MOESM1_ESM.pdf]

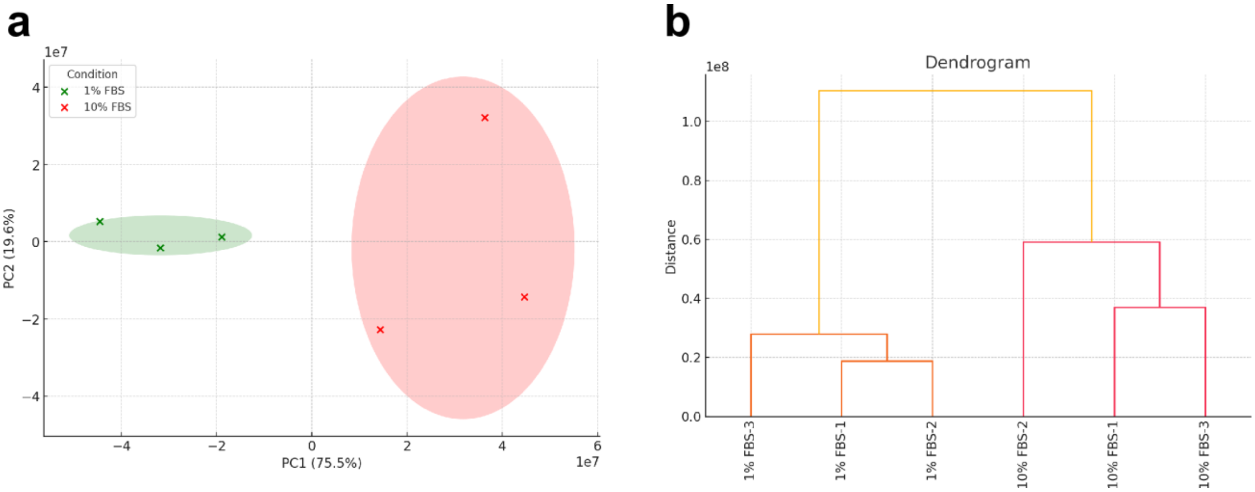

Supplementary Figure 1. Metabolite profile differences in host cells grown under 1% and 10% FBS conditions.

(a) PCA plot displaying the separation of metabolite profiles between host cells grown in 1% FBS (green) and 10% FBS (red). Each point represents an individual sample, with the principal components capturing the variation in the dataset. The clear separation of the two groups indicates distinct metabolomic changes due to the different FBS concentrations. (b) Hierarchical clustering dendrogram showing the relationship among samples based on their metabolite profiles. Samples grown in 1% FBS cluster separately from those grown in 10% FBS, supporting the PCA findings of metabolic divergence under the two conditions.

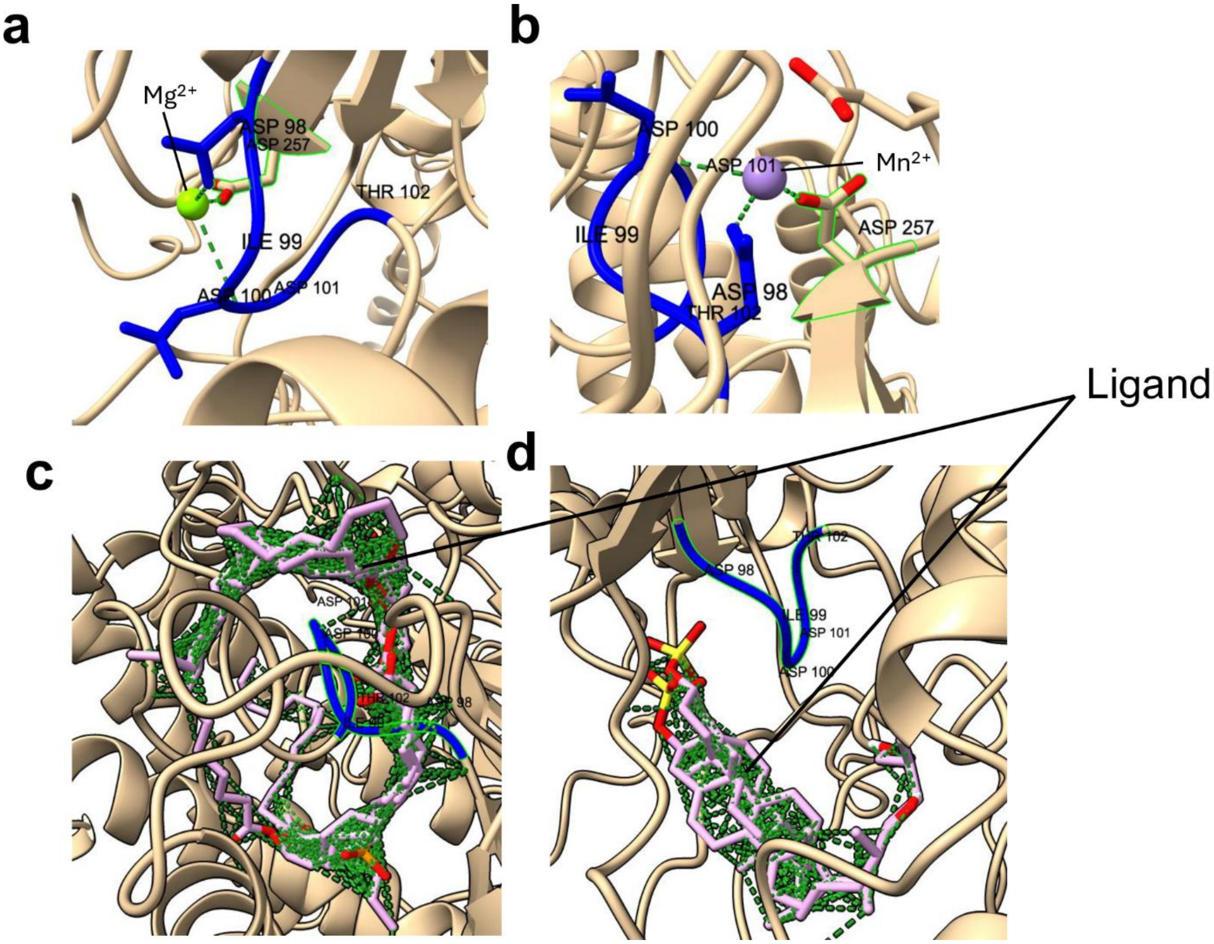

Supplementary Figure 2. **Structural insights into GRA39 and its DxDxT/V catalytic motif.**

(a) Structural view of the GRA39 DxDxT/V motif residues bound to magnesium, with polar contacts indicated by green dashes. **(b)** Close-up view of the key residue interactions in the active catalytic site with manganese bound, highlighting the structural rearrangement. **(c)** Docking of PA to GRA39 by AutoDockVina, showing direct interactions with the DxDxT/V catalytic motif within the GRA39 binding pocket. **(d)** Docking of cholesterol as a non-substrate lipid control. ASP: Aspartic acid, ILE: Isoleucine, THR: Threonine.

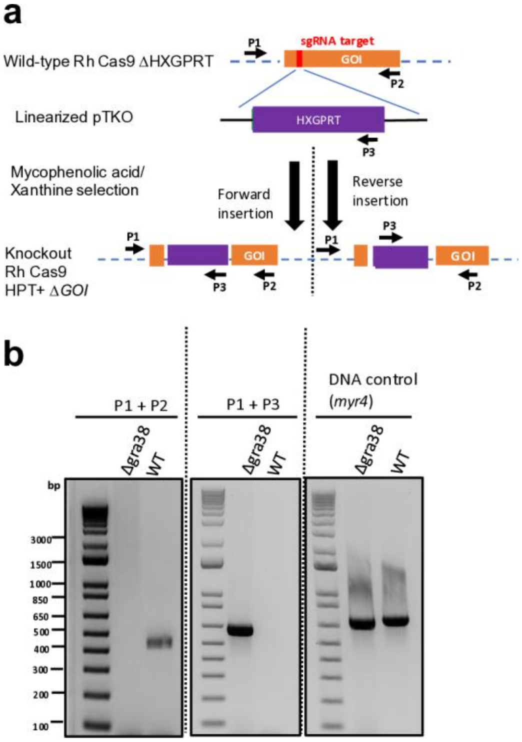

Supplementary Figure 3. Generation of the *GRA38* knockout parasite strain.

(a) Schematic diagram illustrating the strategy used to delete *GRA38* in the type 1 (RH) strain. The CRISPR/Cas9-targeting site is highlighted by a red box. A linearized pTKO plasmid carrying an *HXGPRT* selection cassette served as the repair template; selection was performed using mycophenolic acid and xanthine. **(b)** Confirmation of gene-of-interest (GOI) disruption was performed using primers P1 and P2, which amplify a region within the GOI, with *MYR4* serving as a PCR control. Successful insertion of the repair template was verified using primers P1 and P3.

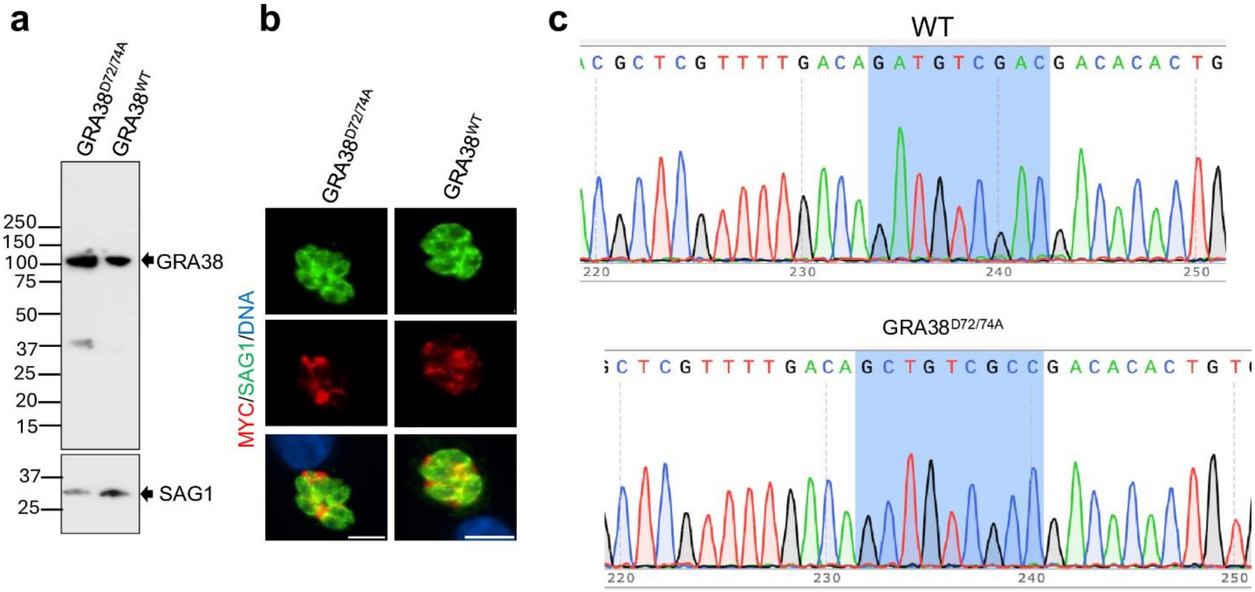

Supplementary Figure 4. Confirmation of complemented strains.

(a) Western blot analysis showing complementation of the *Δgra38* knockout in the type 1 (RH) background with either full-length or inactive GRA38, each expressing a C-terminal MYC tag. The GRA38 protein (117.82 kDa) was detected using an anti-MYC antibody. **(b)** Immunofluorescence assay showing the localization of GRA38 (in red) in complemented *Δgra38* parasites. Scale bar = 6 μM. **(c)** Confirmation of DxDxT/V motif mutations. Genomic DNA was extracted from WT GRA38 or GRA38$^{D72/74A}$ parasites, followed by PCR amplification. Sanger sequencing of the PCR amplicon confirmed mutation of the DxDxT/V motif into AxAxT/V.

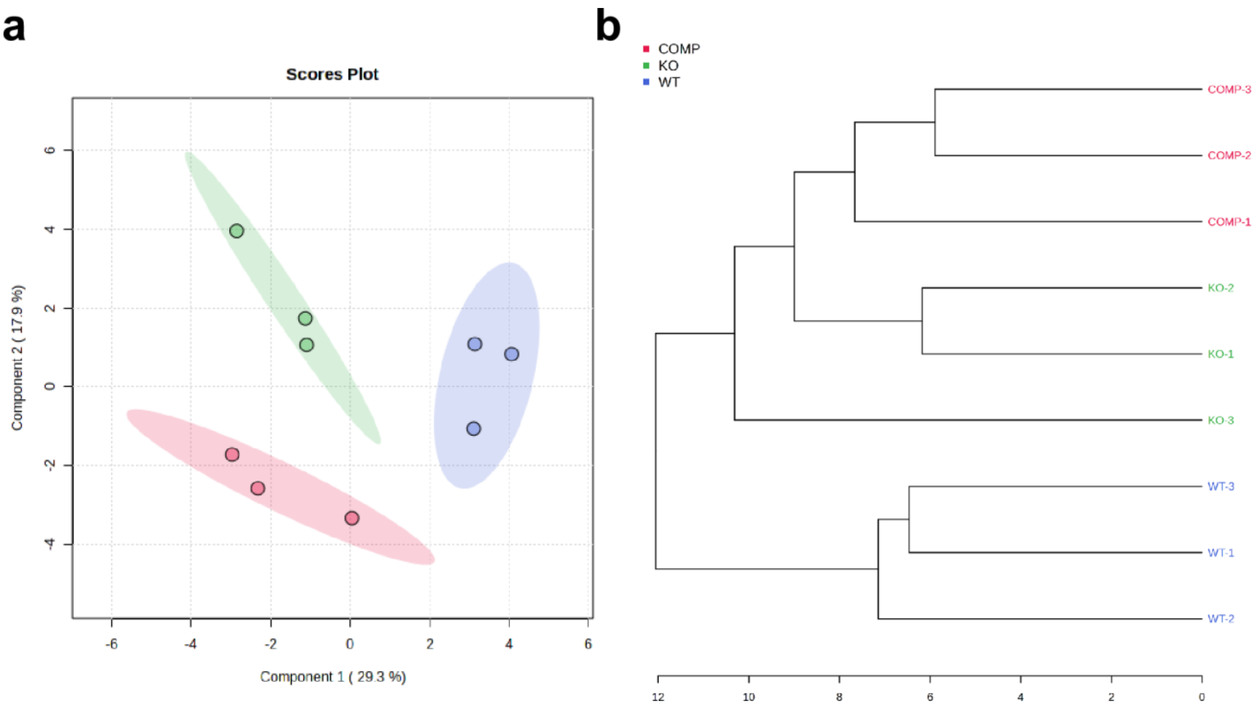

Supplementary Figure 5. Principal component analysis and hierarchical clustering of WT, Δ*gra38*, and complemented parasite strains. (a) PCA derived from the combined data of WT (blue), KO (*Δgra38* knockout, green), and COMP ($GRA38^{WT}$, red). Each point represents a biological replicate. (b) Hierarchical clustering dendrogram showing the relationships among the three experimental groups based on their metabolic profiles. The distance between clusters indicates the similarity among samples.

Figure 8b

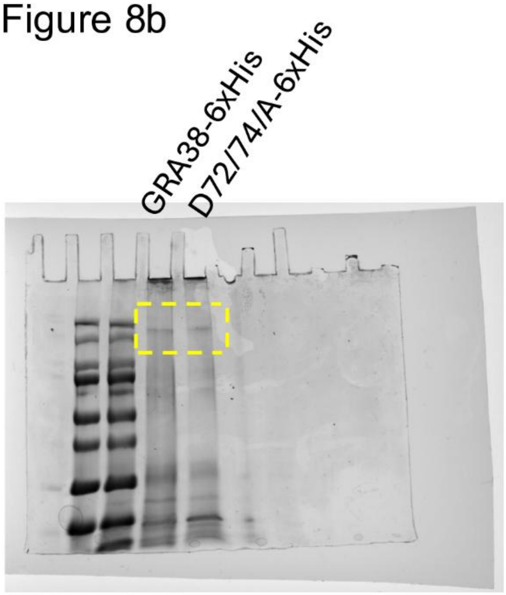

Figure 8c

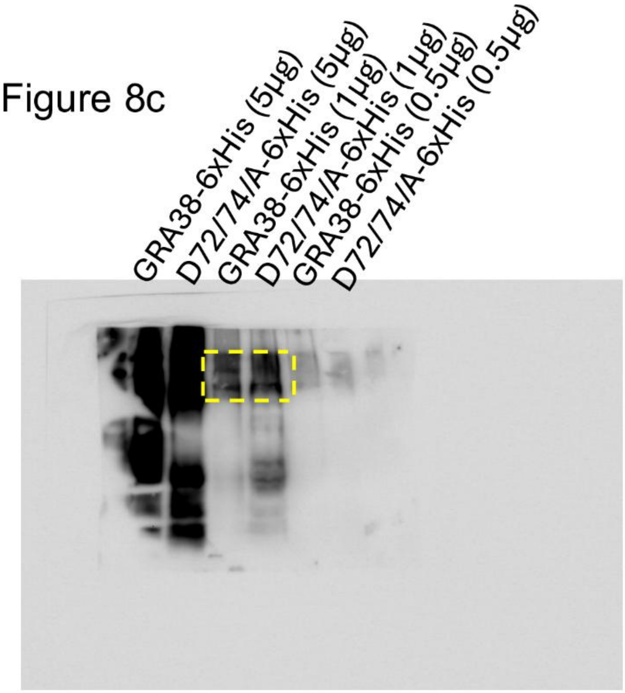

Figure S4a

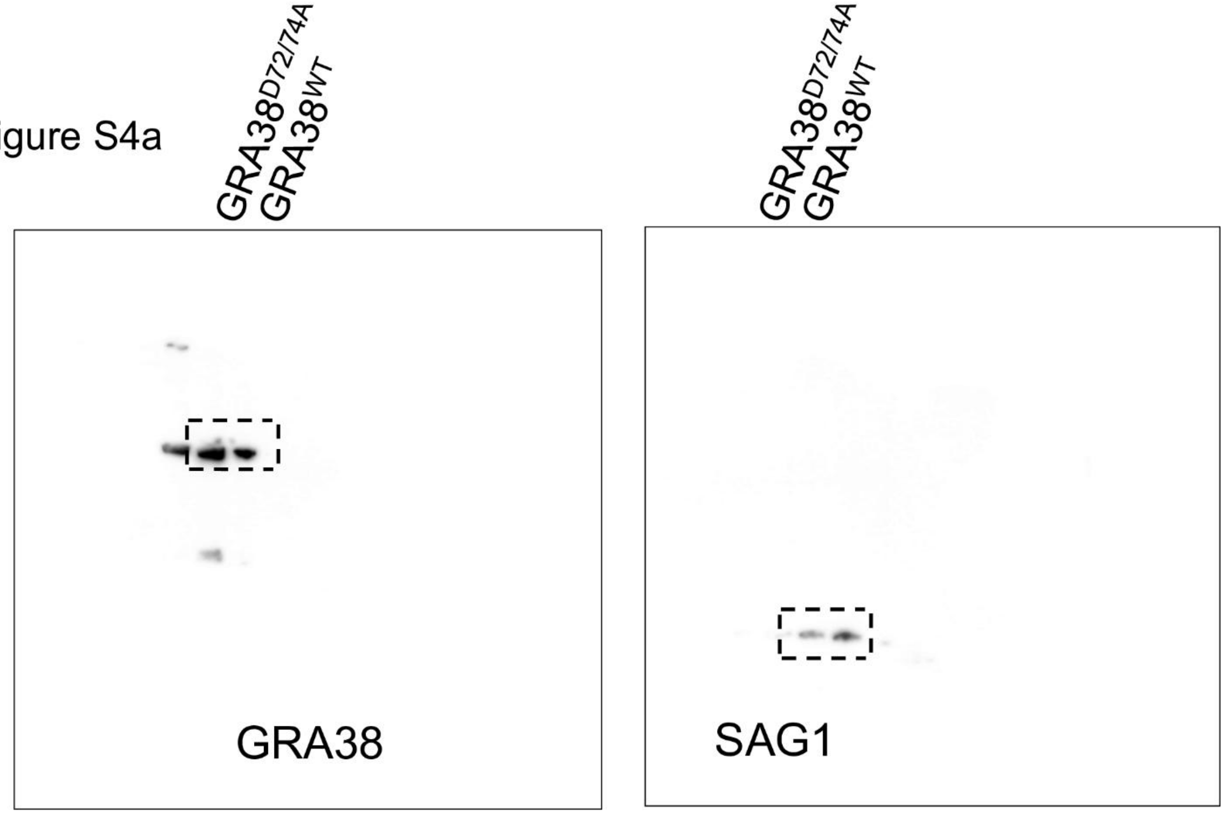

Supplementary Figure 6. All uncropped Western blot images from the study
