# Supplementary material for: A genome-wide CRISPR screen identifies GRA38 as a key regulator of lipid homeostasis during Toxoplasma gondii adaptation to lipid-rich conditions
Source: Nat Commun. 2025 Dec 17;16:11177. doi: 10.1038/s41467-025-66137-5 (PMC12711892; doi:10.1038/s41467-025-66137-5)
Supplement: Supplementary file 2 — Description of Additional Supplementary Files [file 41467_2025_66137_MOESM2_ESM.pdf]

**Supplementary Data 1. Lipidomics Data**

This table contains two sheets. The first sheet presents lipid species abundance between human foreskin fibroblasts (HFFs) cultured in 1% versus 10% fetal bovine serum (FBS). Each row represents a detected lipid species identified by its retention time and m/z value. Annotation refers to the putative lipid identity based on spectral matching or accurate mass and retention time. The assay column indicates the ionization mode used (negLipids = negative ion mode; posLipids = positive ion mode). Species denotes the ion adduct observed. InChIKey provides the standardized chemical identifier. Columns labeled HFFs-1/2/3 1% FBS and HFFs-1/2/3 10% FBS contain raw peak height values from three biological replicates cultured in 1% or 10% fetal bovine serum, respectively. The T-test column indicates the p-value from a two-tailed Student's t-test comparing lipid peak heights between 1% and 10% FBS. L2FC (1% vs. 10%) reports the $\log_2$ fold change in lipid abundance under low serum (1% FBS) relative to high serum (10% FBS) conditions. Negative values indicate lower abundance in 1% FBS.

The second sheet contains *Toxoplasma* lipidomics data comparing wild-type (WT), GRA38 knockout (GRA38KO), and complemented (GRA38COMP) parasites.

**Supplementary Data 2. CRISPR screen data identifying *Toxoplasma gondii* candidate genes with differential fitness under low- and high-serum conditions.**

This table contains results from a genome-wide CRISPR/Cas9 screen comparing *Toxoplasma* fitness in human foreskin fibroblasts (HFFs) cultured in 1% vs. 10% fetal bovine serum (FBS). Each row represents a gene annotated with its ToxoDB ID, description, and additional functional information if available. Fitness scores from individual replicates (P4, P5, and P8; Exp1 and Exp2) under 1% and 10% FBS are provided, along with the calculated phenotype (10%-1%), the number of high-quality sgRNAs supporting each phenotype, and MAGeCK's robust-rank-aggregation ("pos|rank" or "neg|rank"). Mean values are also included. This dataset was used to identify candidate genes that influence parasite fitness in response to lipid availability (Table 1). The raw count numbers for the number of reads for each sgRNA are provided in a separate sheet.

**Supplementary Data 3. Primers and antibodies used in this study.**

These data contain all primers and antibodies used in the study. It includes primers used for Illumina sequencing of sgRNAs amplified from *Toxoplasma* genomic DNA, as well as oligonucleotides designed for gene complementation and endogenous tagging. Additional primers used for site-directed mutagenesis and other molecular biology applications are also included. Antibodies used in immunofluorescence and immunoblotting experiments are listed with corresponding details.
