# Supplementary material for: A genome-wide CRISPR screen identifies GRA38 as a key regulator of lipid homeostasis during Toxoplasma gondii adaptation to lipid-rich conditions
Source: Nat Commun. 2025 Dec 17;16:11177. doi: 10.1038/s41467-025-66137-5 (PMC12711892; doi:10.1038/s41467-025-66137-5)
Supplement: Supplementary file 6 — Reporting Summary [file 41467_2025_66137_MOESM6_ESM.pdf]

nature portfolio

Corresponding author(s): Jeroen Saeij

Last updated by author(s): Oct 20, 2025

Reporting Summary

Nature Portfolio wishes to improve the reproducibility of the work that we publish. This form provides structure for consistency and transparency in reporting. For further information on Nature Portfolio policies, see our Editorial Policies and the Editorial Policy Checklist.

Statistics

For all statistical analyses, confirm that the following items are present in the figure legend, table legend, main text, or Methods section.

n/a	Confirmed	
☐	☒	The exact sample size (*n*) for each experimental group/condition, given as a discrete number and unit of measurement
☐	☒	A statement on whether measurements were taken from distinct samples or whether the same sample was measured repeatedly
☐	☒	The statistical test(s) used AND whether they are one- or two-sided<br>*Only common tests should be described solely by name; describe more complex techniques in the Methods section.*
☒	☐	A description of all covariates tested
☐	☒	A description of any assumptions or corrections, such as tests of normality and adjustment for multiple comparisons
☐	☒	A full description of the statistical parameters including central tendency (e.g. means) or other basic estimates (e.g. regression coefficient) AND variation (e.g. standard deviation) or associated estimates of uncertainty (e.g. confidence intervals)
☐	☒	For null hypothesis testing, the test statistic (e.g. *F*, *t*, *r*) with confidence intervals, effect sizes, degrees of freedom and *P* value noted<br>*Give P values as exact values whenever suitable.*
☒	☐	For Bayesian analysis, information on the choice of priors and Markov chain Monte Carlo settings
☒	☐	For hierarchical and complex designs, identification of the appropriate level for tests and full reporting of outcomes
☐	☒	Estimates of effect sizes (e.g. Cohen's *d*, Pearson's *r*), indicating how they were calculated

Our web collection on statistics for biologists contains articles on many of the points above.

Software and code

Policy information about availability of computer code

Data collection	No software was used to collect the data in this study. Data were collected using standard laboratory protocols and instrument hardware, including Illumina NEXTseq for sequencing sgRNAs in the CRISPR screen, Agilent 1290 UHPLC/Sciex TripleTOF 6600 for lipidomics mass spectrometry, Nikon TE2000 microscope equipped with Hamamatsu ORCA-ER camera for imaging in plaque/invasion/replication assays, and NanoDrop 2000c spectrophotometer for protein concentration measurements. No versions of any associated instrument software are specified. Mouse virulence data were gathered by manual monitoring of survival post-infection. See Methods section for detailed protocols.
Data analysis	1. Custom scripts were used for CRISPR screen analysis (see references# 45 and #58). Microsoft Excel and R (www.R-project.org) were used for statistical analysis of CRISPR data.<br>2. MAGeCK was used to compare raw read counts and calculate p-values for gene selection.<br>3. ImageJ was used to measure plaque areas.<br>4. GraphPad Prism 10 was used for statistical analyses including one-way ANOVA with Tukey's multiple comparisons, two-way ANOVA with Dunnett's or Tukey's multiple comparisons, repeated measures ANOVA with Holm-Šídák's multiple comparisons, and log-rank (Mantel-Cox) tests.<br>5. MS-DIAL (version 4.9) was used to process LC-MS/MS data for lipidomics, including peak detection, alignment, MS2 deconvolution, adduct identification, blank subtraction, gap filling, and annotation.<br>6. ChromaTOF and the metabolomics BinBase database were used to process GC-TOF MS data for lipidomics.<br>7. MetaboAnalyst (version 6.0) was used for principal component analysis (PCA), dendrograms, volcano plots, t-tests, and ANOVA in lipidomics.<br>8. AlphaFold 3 was used for protein structure prediction.<br>9. Foldseek was used for protein structure search.

10. AutoDock Vina (version 1.2.0) and SwissDock 2024 were used for molecular docking.
11. FoldMason was used for multiple protein structure alignment.
12. NanoDrop 2000c software was used for protein concentration measurements. See Methods section for details.

For manuscripts utilizing custom algorithms or software that are central to the research but not yet described in published literature, software must be made available to editors and reviewers. We strongly encourage code deposition in a community repository (e.g. GitHub). See the Nature Portfolio guidelines for submitting code & software for further information.

Data

Policy information about availability of data

All manuscripts must include a data availability statement. This statement should provide the following information, where applicable:

- Accession codes, unique identifiers, or web links for publicly available datasets
- A description of any restrictions on data availability
- For clinical datasets or third party data, please ensure that the statement adheres to our policy

All data supporting the findings of this study are available within the paper and its Supplementary Information, as well as in the Source Data file provided with this manuscript.

Research involving human participants, their data, or biological material

Policy information about studies with human participants or human data. See also policy information about sex, gender (identity/presentation), and sexual orientation and race, ethnicity and racism.

Reporting on sex and gender	Not applicable. This study did not involve human participants, human data, or human biological material.
Reporting on race, ethnicity, or other socially relevant groupings	Not applicable. This study did not involve human participants, human data, or human biological material.
Population characteristics	Not applicable. This study did not involve human participants, human data, or human biological material.
Recruitment	Not applicable. This study did not involve human participants, human data, or human biological material.
Ethics oversight	Not applicable. This study did not involve human participants, human data, or human biological material. Ethics oversight was provided for animal experiments involving mice, as detailed in the Methods section (e.g., approval by the University of California Davis Institutional Animal Care and Use Committee).

Note that full information on the approval of the study protocol must also be provided in the manuscript.

Field-specific reporting

Please select the one below that is the best fit for your research. If you are not sure, read the appropriate sections before making your selection.

[x] Life sciences [ ] Behavioural & social sciences [ ] Ecological, evolutionary & environmental sciences

For a reference copy of the document with all sections, see nature.com/documents/nr-reporting-summary-flat.pdf

Life sciences study design

All studies must disclose on these points even when the disclosure is negative.

Sample size	No statistical method was used to predetermine sample sizes, but sample sizes were determined based on standard practices in the field to ensure adequate statistical power.
Data exclusions	No data were excluded from the analyses. All collected data from CRISPR screens, lipidomics, growth assays, and mouse experiments were included. We didn't have pre-established exclusion criteria.
Replication	All experiments were performed with at least three independent biological replicates to ensure reproducibility, as detailed in the Methods section. Mouse experiments were conducted with single cohorts but included multiple animals per group (n=5) to validate findings. Replication success is confirmed by consistent results across replicates, as shown in figure legends.
Randomization	Randomization was not explicitly applied to in vitro experiments (e.g., CRISPR screen, lipidomics) due to controlled laboratory conditions where samples were assigned based on experimental design (e.g., 1% vs. 10% FBS). For mouse virulence experiments, animals were randomly assigned to control or experimental groups to minimize bias, as described in the Methods section.
Blinding	Blinding was not performed for some in vitro experiments (e.g., CRISPR screen, lipidomics) due to the objective nature of the measurements (e.g., sequencing read counts, mass spectrometry data). For other experiments, blinding was implemented with investigators unaware of group assignments until analysis was complete.

Reporting for specific materials, systems and methods

We require information from authors about some types of materials, experimental systems and methods used in many studies. Here, indicate whether each material, system or method listed is relevant to your study. If you are not sure if a list item applies to your research, read the appropriate section before selecting a response.

Materials & experimental systems

n/a	Involved in the study
☐	☒ Antibodies
☐	☒ Eukaryotic cell lines
☒	☐ Palaeontology and archaeology
☐	☒ Animals and other organisms
☒	☐ Clinical data
☒	☐ Dual use research of concern
☒	☐ Plants

Methods

n/a	Involved in the study
☒	☐ ChIP-seq
☒	☐ Flow cytometry
☒	☐ MRI-based neuroimaging

Antibodies

Antibodies used

1. Anti-SAG1 (Mouse monoclonal), generously provided by Dr. John C. Boothroyd, 1:100 dilution
2. Anti-Mouse Alexa Fluor 488 (goat polyclonal), Thermo Fisher, Cat#A11029, (1:3000) dilution
3. Anti-Mouse Alexa Fluor 594 (goat polyclonal), Thermo Fisher, Cat#A11032, (1:3000) dilution
4. Anti-Rat Alexa Fluor 594 (goat polyclonal), Thermo Fisher, Cat#A11007, (1:3000) dilution
5. Anti-Rabbit Alexa Fluor 488 (goat polyclonal), Thermo Fisher, Cat#A11008, (1:3000) dilution
6. Anti-Rabbit Alexa Fluor 594 (goat polyclonal), Thermo Fisher, Cat#A11037, (1:3000) dilution
7. Anti-GRA5 (Mouse monoclonal), BioVision, Cat#A1299, (1:500) dilution
8. Goat anti-rabbit HRP, Jackson ImmunoResearch Laboratories Inc, Cat#111-035-003, 1:10000 dilution
9. Goat anti-rat HRP, Jackson ImmunoResearch Laboratories Inc, Cat#112-035-003, 1:10000 dilution
10. Mouse anti-MYC, Cell signaling, CAT#2276, 1:1000 dilution
11. Rabbit anti-GRA7, generously provided by Dr. John C. Boothroyd, (1:5000) dilution
12. Anti-IMC1 (mouse), generously provided by Dr. Gary Ward, (1:3000) dilution
13. 6x-His Tag Monoclonal Antibody, Thermo Fisher, Cat#MA1-21315, (1:2000) dilution

Validation

2. https://www.thermofisher.com/antibody/product/Goat-anti-Mouse-IgG-H-L-Highly-Cross-Adsorbed-Secondary-Antibody-Polyclonal/A-11029
3. https://www.thermofisher.com/antibody/product/Goat-anti-Mouse-IgG-H-L-Highly-Cross-Adsorbed-Secondary-Antibody-Polyclonal/A-11032
4. https://www.thermofisher.com/antibody/product/Goat-anti-Rat-IgG-H-L-Cross-Adsorbed-Secondary-Antibody-Polyclonal/A-11007
5. https://www.thermofisher.com/antibody/product/Goat-anti-Rabbit-IgG-H-L-Cross-Adsorbed-Secondary-Antibody-Polyclonal/A-11008
6. https://www.thermofisher.com/antibody/product/Goat-anti-Rabbit-IgG-H-L-Highly-Cross-Adsorbed-Secondary-Antibody-Polyclonal/A-11037
7. The BioVision antibody for Anti-GRA5, clone TG-17.113, with former catalog number A1299, has been transitioned to Abcam, an affiliated company. The new catalog number for this product is ab286170.
https://www.abcam.com/en-us/products/primary-antibodies/gra5-antibody-tg-17113-ab286170
8. https://www.jacksonimmuno.com/catalog/products/111-035-003
9. https://www.jacksonimmuno.com/catalog/products/112-035-003
10. https://www.cellsignal.com/products/primary-antibodies/myc-tag-9b11-mouse-mab/2276?srsltid=AfmBOorrODA0BF37xaOZh7VXF3uDfRPavphMSCnr4yh0K3Vjm6k3TrcW
13. https://www.thermofisher.com/antibody/product/6x-His-Tag-Antibody-clone-HIS-H8-Monoclonal/MA1-21315

Eukaryotic cell lines

Policy information about cell lines and Sex and Gender in Research

Cell line source(s)

The study utilized Human Foreskin Fibroblasts (HFF) as the primary eukaryotic cell line for culturing Toxoplasma gondii. HFF cells were originally obtained from Dr. John Boothroyd and originated from a pool of anonymous donors.

Authentication

HFF cells were authenticated by morphological examination and growth characteristics consistent with ATCC standards.

Mycoplasma contamination

All HFF cell lines were routinely tested for mycoplasma contamination using a PCR-based detection method or commercial kit. Cells were confirmed to be mycoplasma-free throughout the study.

Commonly misidentified lines (See ICLAC register)	The study used HFFs as the primary eukaryotic cell line for culturing Toxoplasma gondii. HFF cells were checked against the ICLAC Register of Misidentified Cell Lines (available at iclac.org/databases/cross-contaminations/). HFF is not listed as a misidentified cell line in the current register (version 13, last updated April 26, 2024, with 593 entries). No other cell lines were used in this study.

Animals and other research organisms

Policy information about studies involving animals; ARRIVE guidelines recommended for reporting animal research, and Sex and Gender in Research

Laboratory animals	For laboratory animals, the study involved mice. The strain was CD-1 (outbred sourced from Charles River Labs), and the age was 6 weeks at the start of experiments
Wild animals	The study did not involve wild animals. No animals were observed in or captured in the field, and no field-collected samples were used.
Reporting on sex	Findings apply to only one sex; the study used female CD-1 strain. Sex was considered in the study design, with all mice being female as confirmed by breeder documentation and visual inspection. Data disaggregated by sex are not provided, as all experiments used 6-week-old female mice to standardize conditions. Sex-based analyses were not performed, as the focus was on parasite virulence rather than sex-specific effects. the study aimed to assess baseline virulence in a controlled model, and sex differences were not a research objective.
Field-collected samples	The study did not involve samples collected from the field. All laboratory work was conducted with cultured Toxoplasma gondii in HFF and laboratory bred 6-week-old female CD-1 mice under controlled conditions.
Ethics oversight	The organization(s) that approved or provided guidance on the study protocol was the University of California Davis Institutional Animal Care and Use Committee (IACUC). Ethical approval was required and obtained. Animal experiments were approved by the Institutional Animal Care and Use Committee (IACUC) at the University of California, Davis with protocol number #23860.

Note that full information on the approval of the study protocol must also be provided in the manuscript.

Plants

Seed stocks	The study did not involve plants. No plant species or materials were used in the research
Novel plant genotypes	Not applicable. The study did not involve seed stocks or any plant materials, and no collection or generation of seed stocks occurred
Authentication	Not applicable. The study did not involve the generation or authentication of novel plant genotypes
